# Perioperative amino acid infusion reestablishes muscle net balance during total hip arthroplasty

**DOI:** 10.14814/phy2.15055

**Published:** 2021-09-23

**Authors:** David D. Church, Scott E. Schutzler, Robert R. Wolfe, Arny A. Ferrando

**Affiliations:** ^1^ Department of Geriatrics Center for Translational Research in Aging & Longevity Donald W. Reynolds Institute on Aging University of Arkansas for Medical Sciences Little Rock Arkansas USA

**Keywords:** amino acids, anabolism, skeletal muscle, stable isotope tracer

## Abstract

Surgery and anesthesia induce a catabolic response that leads to skeletal muscle protein loss. Previous investigations have observed positive effects of perioperative nutrition. Furthermore, the benefits of exogenous amino acids on muscle protein kinetics are well established. However, no investigation has focused on muscle protein kinetics with and without perioperative amino acid infusion. Thus, we aimed to assess the effect of perioperative amino acid (AA) infusion on muscle protein balance in individuals undergoing elective total hip arthroplasty (THA). Elective THA patients were randomized to undergo a metabolic study prior to surgery (*n* = 5; control [CON]), intraoperative AA infusion (*n* = 9), or no AA (*n* = 13; standard of care [SC]). The CON group was studied prior to surgery to provide nonoperative/non‐anesthesia muscle protein kinetic reference values. The bolus infusion method with ^13^C_6_‐phenylalanine injected at time 0, and [^15^N]‐phenylalanine 30 min later was used to calculate muscle protein synthesis (MPS), protein breakdown (MPB), and net balance (MPS−MPB). Perioperative AA significantly improved muscle net balance as compared to SC (−0.005 ± 0.018%/h vs. −0.052 ± 0.011%/h) but not CON (0.003 ± 0.013%/h). The AA infusion significantly increased muscle net balance via a significant increase in MPS (AA = 0.062 ± 0.007%/h; SC = 0.037 ± 0.004%/h; CON = 0.072% ± 0.005%/h), and a nonsignificant attenuation of MPB (AA = 0.067 ± 0.012%/h; SC = 0.089 ± 0.014%/h; CON = 0.075 ± 0.011%/h). Our data support the use of perioperative AA infusion during elective THA as pragmatic strategy to offset the loss of surgically induced skeletal muscle protein.

**New and Noteworthy**

Our research evaluates the efficacy of perioperative (total hip and knee arthroplasty) amino acid infusion to offset surgery‐induced muscle catabolism as compared to pre‐ and perioperative controls. Perioperative amino acid infusion restores muscle anabolism to levels that mirrored preoperative controls, but superior to perioperative controls not receiving amino acid infusion. Collectively, perioperative amino acid infusion effectively restores muscle net balance to zero.

## 1 INTRODUCTION

By the year 2040, the demand for total hip arthroplasty (THA) is projected to increase to 1.4 million patients (Singh et al., 2019). THA is often a last resort procedure for patients with end‐stage osteoarthritis and, in most cases, THA significantly improves pain, mobility function, and quality of life (Bamman et al., 2015; Ninomiya et al., 2020; Singh et al., 2019; Yoshiko et al., 2020). However, lower body muscle strength and functional performance of THA patients can remain significantly lower than healthy older adults several years after surgery (Ninomiya et al., 2020; Yoshiko et al., 2020). Impairments in muscle mass and strength following THA can lead to instability during walking and abnormal gait patterns, increasing the chance for falls and reduced quality of life (Ninomiya et al., 2020). Given that the requirement for THA is increasing exponentially, attenuating these impairments is a major public health problem that requires further attention.

Surgical stress has been well‐documented to promote a catabolic physiological state. Surgery‐induced catabolism accelerates skeletal muscle protein breakdown (MPB) to supply amino acid precursors for wound healing, immune function, and carbon skeletons for gluconeogenesis (Church et al., 2019). Skeletal muscle protein is the body's primary labile endogenous source of amino acids; however, the tissue's primary responsibility is work/locomotion. Thus, strategies that attenuate skeletal muscle protein loss during surgery are essential to reduce recovery time and improve quality of life. One such strategy is leveraging perioperative amino acid infusion to spare skeletal muscle protein by providing the body with an exogenous source of amino acids. Previous research has indicated this to be an effective strategy to improve whole‐body anabolism in humans (Donatelli et al., 2006) and muscle anabolism in murine models (Yamaoka et al., 2006). However, to our knowledge no investigations have assessed the effects on human muscle protein. Therefore, the purpose of this study was to investigate the effectiveness of a perioperative amino acid infusion on mitigating the net breakdown of skeletal muscle during surgery in THA patients.

## 2 METHODS

### 2.1 Patients

THA patients were recruited from the University of Texas Medical Branch (UTMB) or University or Arkansas for Medical Sciences (UAMS) scheduled for elective THA (total hip, *n* = 21; resurfacing, *n* = 11). Patients were randomly assigned to receive amino acid (*n* = 14; AA) infusion during surgery or standard of care treatment/surgery (*n* = 14; SC). An additional subset of patients was studied prior to surgery (*n* = 5; CON) to characterize muscle protein kinetics in the absence of surgical stress. Patients were excluded if they were taking insulin, thiazolidinedione drugs, or metformin; had a history of chronic renal insufficiency/disease or liver disease; had uncontrolled hypertension at the time of presurgical screening; had any history of hypo‐ or hypercoagulation disorders including taking of coumadin; had a history of atrial fibrillation, angina, or congestive heart failure; had recently (6 mo or less) been treated for cancer other than basal cell carcinoma; or if they were pregnant. UTMB/UAMS Institutional Review Board‐approved informed consent was obtained by the study nurse after the study was described and discussed in detail.

### 2.2 Perioperative infusion study

For THA patients, a 1‐h perioperative metabolic study was conducted to determine skeletal muscle protein fractional synthetic (FSR) and fractional breakdown (FBR) rates in the surgical limb. Patients in the AA condition received a primed (0.45 ml) continuous infusion (1.35 ml/h/kg) of 10% Travasol (Clintec Nutrition, Deerfield, IL). The concentrations (mg/ml and µmol/ml, respectively) of the Travasol (as reported by the manufacturer) were: leucine 7.3, 55.6; isoleucine 6, 45.7; lysine 5.8, 39.7; valine 5.8, 49.5; phenylalanine 5.6, 33.9; histidine 4.8, 30.9; threonine 4.2, 35.3; methionine 4, 26.8; tryptophan 1.8, 8.8; alanine 20.7, 232.3; arginine 11.5, 66.0; glycine 10.3, 137.2; proline 6.8, 59.1; serine 5, 47.6; tyrosine 0.4, 2.2; total amino acids 100 mg/ml, and total nitrogen 16.5 mg/ml. The method for determining FSR and FBR has been validated and described in detail (Zhang et al., 2002). Briefly, this method involves a stable isotope bolus tracer injection and the measurement of enrichment in arterial blood samples and muscle biopsies. Calculations of FSR and FBR are based on the precursor‐product principle (see below). Patients were anesthetized using a standardized anesthesia protocol of propofol, isoflurane, and an opioid of choice as deemed necessary by the anesthesiologist. Blood samples were drawn from an existing radial arterial line utilized for surgical monitoring. Isotope infusions were given via an existing catheter in the antecubital vein. A background blood sample was drawn before the isotope was injected. Once the surgeon had the patient prepped and draped for surgery, the stable isotope L‐ring‐[^13^C_6_]‐phenylalanine (35 µmol/kg) was given by an intravenous push and the study timer was started. Blood samples were drawn at ~5, 10, 15, 20, 30, 35, 40, 50, and 60 min on the timer (exact times were recorded and used for calculations). Samples of gluteus maximus (~100–200 mg) were taken 5 and 60 min after tracer injection, subsequently cleaned of visible fat, and snap frozen in liquid nitrogen. To measure FBR, a second tracer, [^15^N]‐phenylalanine (35 µmol/kg), was injected at the 30 min time point and arterial blood was sampled as described above.

### 2.3 Analytical methods

Blood and muscle samples were analyzed for isotope enrichment using methods previously described (Park et al., 2021). Briefly, upon thawing, biopsy samples were precipitated with 800 µl of 14% perchloroacetic acid.

Tissue was homogenized and centrifuged, and tissue‐free amino acids (and labeled phenylalanine) were extracted from the supernatant by cation exchange chromatography (Dowex AG 50W‐8X; 100‐ to 200‐mesh H+ form; Bio‐Rad Laboratories, Richmond, CA) and dried under vacuum (Savant Industries, Farmingdale, NY). The remaining muscle pellet was washed and dried and hydrolyzed in 6 N HCl at 50℃ for 24 h. Muscle free and protein bound phenylalanine enrichment was determined using the *tert*‐butyldimethylsilyl derivative and GC‐MS (Hewlett‐Packard 5980/5989B) with electron impact ionization and selective ion monitoring for ions 234, 235, 239, and 240. The enrichments were corrected for the contribution of the abundance of isotopomers of lower weight to the apparent enrichment of isotopomers with larger weight. A skew correction factor was also used to calculate L‐ring‐[^13^C_6_]‐phenylalanine enrichment in the blood and muscle supernatant (Rosenblatt et al., 1992).

### 2.4 Fractional synthetic and breakdown rate calculations

The precursor‐product FSR and FBR were directly calculated from the enrichment decay over time after the tracer bolus (Zhang et al., 2002). FSR uses the change in enrichment in tracer bound to muscle proteins divided by the change in intracellular enrichment in muscle over the study period (Equation 1):

(1) $$FSR=\frac{E_{B}\left(t_{3}\right)‐E_{B}\left(t_{1}\right)}{\int_{t_{1}}^{t_{3}}E_{F}\left(t\right)dt}$$

where E_F_ and E_B_ are the enrichments in the free intracellular and bound amino acid, respectively, pool in muscle, *t_1_* is the time 0 min, and *t_3_* is the time 60 min.

Calculations of FBR are also based on the precursor‐product principle; however, the precursor is now the unlabeled protein bound amino acid (phenylalanine), and the product the arterial dilution of L‐[^15^N]‐phenylalanine (Equation 2):

(2) $$FBR=\frac{\left[E_{M}\left(t_{2}\right)‐E_{M}\left(t_{1}\right)\right]\;\int_{t_{2}}^{t_{3}}\left[E_{A}\left(t\right)‐E_{M}\left(t\right)\right]dt‐\left[E_{M}\left(t_{3}\right)‐E_{M}\left(t_{2}\right)\right]\;\left[\int_{t_{1}}^{t_{2}}E_{A}\left(t\right)‐E_{M}\left(t\right)dt\right]}{\int_{t_{2}}^{t_{3}}E_{M}\left(t\right)dt\;\int_{t_{1}}^{t_{2}}E_{A}\left(t\right)dt‐\int_{t_{1}}^{t_{2}}E_{M}\left(t\right)dt\;\int_{t_{2}}^{t_{3}}E_{A}\left(t\right)dt}\;\left(\frac{Q_{M}}{T}\right)$$

where E_m_ is the enrichment in the muscle intracellular free pool, E_A_ is the enrichment in the arterial blood, *t_2_* is the time 30 min, and Q_m_/T is the ratio of free to bound Phe in muscle. Muscle net balance was calculated by subtracting FBR from FSR. Therefore, a positive muscle net balance would indicate anabolism, whereas a negative value would indicate catabolism. FSR and FBR results are reported in units of %/h.

### 2.5 Statistics

Outliers were assessed on the primary outcome variable, muscle net balance. One outlier in the AA group had a muscle net balance value of more than three standard deviations away from the group mean. In addition, for three patients only FSR data were available, one patient withdrew from the study, and one patient group (AA or SC) could not be discerned. After removal of these patient's data, normality of distribution of variables was tested using the Shapiro–Wilk test. A one‐way analysis of variance was used to assess significant differences between the CON, SC, and AA groups. Significant main effects were followed up with Tukey's post hoc testing. Cohen's D was used to assess post hoc effect sizes for muscle FSR, FBR, and NB. Results are expressed as means ± SEM (95% confidence interval). An alpha level of *p* < 0.05 was considered statistically significant. Statistical analyses were performed with IBM SPSS software (version 26; IBM Corp. Armonk, NY, USA).

## 3 RESULTS

Demographics for patients who completed the study are presented in Table 1. The one‐way ANOVA indicated no significant main effects between groups for all demographic variables.

**TABLE 1:** Patient demographics

Group	*N* (m/f)	Age (years)	Height (M)	Total mass (kg)	BMI (kg/m^2^)
Control	5 (4/1)	53.0 ± 5.5	1.76 ± 0.03	105.0 ± 2.9	33.8 ± 0.5
Standard of care	13 (7/6)	55.5 ± 3.2	1.72 ± 0.03	88.1 ± 5.4	29.5 ± 1.2
Amino acid	9 (7/2)	52.2 ± 2.8	1.74 ± 0.03	85.7 ± 5.4	28.2 ± 1.8

Note

Data presented as mean ± SEM.

Abbreviations: f, female; kg, kilogram; m, male; M, meters.

**FIGURE 1:**
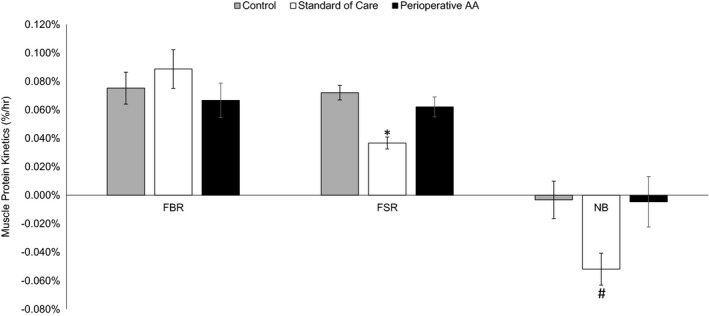
Muscle protein fractional breakdown rate (FBR), fractional synthetic rate (FSR), and muscle net balance (NB) in percent per hour (%/h) in the control, standard of care, and perioperative amino acid (AA) infusion groups. *Indicate standard of care was significantly less than control and perioperative AA. #Indicate significant difference between standard of care and perioperative AA. Values presented as means ± SEM

The one‐way ANOVA indicated a significant main effect for FSR (F = 10.412; *p* = 0.001; η^2^ = 0.465) and muscle net balance (F = 3.752; *p* = 0.038; η^2^ = 0.252). However, no main effect was observed for FBR (F = 0.762; *p* = 0.478; η^2^ = 0.060) between SC (0.0.089 ± 0.014%/h [0.062–0.115%/h]), AA (0.067 ± 0.012%/h [0.043–0.090%/h]), and CON (0.075 ± 0.010%/h [0.053–0.097%/h]). Tukey post hoc testing indicated FSR was significantly attenuated in SC (0.037 ± 0.004%/h [0.028–0.045%/h]) as compared to AA (*p* = 0.006; 0.062 ± 0.007%/h [0.076–0.048%/h]) and CON (*p* = 0.002; 0.066 ± 0.007%/h [0.073–0.057%/h]), however FSR was not significantly different (*p* = 0.549) between AA and CON (Figure 1).

Tukey post hoc testing indicated muscle net balance was significantly (*p* = 0.049) improved in AA (−0.005 ± 0.018%/h [−0.039 to 0.030%/h]) as compared to SC (−0.052 ± 0.011%/h [−0.074 to −0.030%/h]). CON muscle net balance (−0.011 ± 0.013%/h [−0.035 to 0.015%/h]) was not significantly different from AA (*p* = 0.998 [−0.039 to 0.030%/h]) or SC (*p* = 0.107). All post hoc effect sizes are present in Table 2.

**TABLE 2:** Effect size values for fractional breakdown rate (FBR), synthesis rate (FSR), and net balance (NB) compared to presurgical controls and standard of care

Group	Compared to presurgical controls			Compared to standard of care		
	FBR	FSR	NB	FBR	FSR	NB
Amino acid infusion	0.24	0.17	0.11	0.5	1.42	1.03
Standard of care	0.32	2.04	1.12	—	—	—

## 4 DISCUSSION

Our results indicate that surgery reduced muscle net protein balance, and that perioperative amino acid infusion restored muscle net protein balance to presurgery levels primarily through an increase in muscle protein synthesis (MPS). Although amino acid infusion attenuated muscle protein breakdown (MPB) by ~25%, this effect was not statistically significant. To the authors’ knowledge, this is the first study to observe a perioperative improvement in muscle net balance in humans. The effect of AA infusion was robust enough to return perioperative muscle net balance to zero (−0.005%). Further, perioperative amino acid infusion was capable of mitigating the effects of fasting on the efflux of muscle amino acids (Biolo et al., 1997).

The anabolic effect of amino acids on muscle protein has long been established. Exogenous amino acids have been observed to improve muscle net balance via a suppression of MPB and stimulation of MPS (Biolo et al., 1997; Bohé et al., 2003; Tipton et al., 1999, 2007; Volpi et al., 1998). In the postabsorptive state, the MPS rate is lower as the only amino acid precursors appearing in the intracellular space is supplied by MPB. In hypercatabolic states (e.g., burn injury, surgery, sepsis), the rate of MPS is elevated as compared to controls, however, this is due to an increase in amino acids entering the intracellular space from an elevated MPB rate (Biolo et al., 2002; Wolfe, 2018). When exogenous amino acids are provided, the rate of MPB is attenuated as muscle protein is no longer required to supply precursor amino acids for MPS, nor the synthesis of other required proteins in the body. We observed a nonsignificant 25% attenuation of MPB, which is less than our previous findings of a 35% in elderly adults receiving the same infusion rate and profile of amino acids under non‐stress conditions (Volpi et al., 1998). The lower reduction of MPB as compared to our previous investigations is most likely due to the potent catabolic stress that accompanies major surgery and anesthesia (Svedjeholm et al., 1990; Taggart et al., 1991; Wolfe, 2018). Surgical stress induces a catabolic hormonal milieu leading to increased energy expenditure, hyperglycemia, and acute phase protein synthesis (Church et al., 2019; Codère‐Maruyama et al., 2016; Finnerty et al., 2013; Svedjeholm et al., 1990; Wolfe, 2018). In this context, skeletal muscle is no longer the primary site for anabolism, as the demand for amino acids elsewhere in the body takes precedence. In this physiological state, muscle is predominately a reservoir for amino acids to support protein turnover in other tissues. For example, essential amino acid arteriovenous difference and flux across the leg were negative, but positive across the myocardium in patients undergoing elective aortocoronary bypass surgery (Svedjeholm et al., 1990). It is plausible that under surgical conditions, the amino acid infusion rate was not high enough to supply adequate amino acid precursors for both protein synthesis and gluconeogenesis. However, previous work has indicated that the primary mechanism of action of amino acids is increased MPS (Church et al., 2020; Sheffield‐Moore et al., 2000). Despite the attenuated MPB rate not reaching statistical significance, the elevation in MPS was robust enough to effectively prevent the loss of skeletal muscle protein/amino acids.

Perioperative amino acid infusion restored skeletal muscle net balance to essentially zero (−0.005%), primarily through a stimulation in MPS. These results are in agreement with previous studies observing intravenous administration of amino acids elevating MPS in propofol‐anesthetized rats (Yamaoka et al., 2006). To our knowledge, this is the first investigation to assess skeletal muscle net balance during perioperative amino acid infusion in humans; however, previous work focusing on whole‐body protein balance are consistent with our results. For example, perioperative amino acid infusion under general anesthesia, alone or combined with an epidural, significantly improved whole‐body protein balance (Donatelli et al., 2006). Further, patients undergoing elective coronary artery bypass graft surgery demonstrated slightly positive (2.1 ± 5.4 µmol/kg/h) postoperative whole‐body protein balance when amino acids were provided perioperatively (Codère‐Maruyama et al., 2016). In our study, muscle protein kinetics were assessed perioperatively, whereas Codere‐Maruyama (Codère‐Maruyama et al., 2016) studied whole‐body protein kinetics patients postoperatively. Considering skeletal muscle is no longer a priority tissue during surgery, our observation of a zero‐muscle net balance indicates non‐muscle tissue requirements were satisfied. This notion is supported by previous investigations in which whole‐body protein balance is improved without an elevation in MPS during energy deficit (Gwin et al., 2021).

The lack of whole‐body protein balance measures prevents us from speculating how much muscle protein turnover contributed to whole‐body protein turnover during surgery. Nonetheless, Taggert and colleagues (Taggart et al., 1991) demonstrated urinary 3‐methylhistidine was significantly greater 48 h postoperatively compared to 48 h preoperatively, indicating accelerated muscle protein breakdown. Furthermore, previous research using lower intraoperative amino acid infusion rates (0.02 ml of Travasol/kg/min) improved whole‐body protein balance, indicating a sparing of body protein (Donatelli et al., 2006). Taken together, it is plausible that the improvement in muscle net balance we observed is the result of non‐muscle tissue requirements being met through elevations in plasma essential amino acids (Church et al., 2020; Codère‐Maruyama et al., 2016; Svedjeholm et al., 1990; Taggart et al., 1991). Nonetheless, recent work has demonstrated that 20 g of essential amino acids twice a day for 6‐week preoperatively and postoperatively attenuated the loss of muscle volume in older adults recovering from total knee arthroplasty (Dreyer et al., 2018). Furthermore, THA patients receiving 15 g of essential amino acids three times a day significantly improved maximal voluntary contraction of the leg extensors 8 weeks postoperatively as compared to standard of care (Ferrando et al., 2013). Therefore, it is apparent that multiple therapies throughout the surgical process are beneficial for optimal recovery.

The present manuscript is not without limitations. For example, we did not measure any molecular markers of protein synthesis or breakdown explaining our metabolic outcomes. Although amino acid‐induced increases in MPS are well established and likely related to an increase in mammalian target of rapamycin complex 1 signaling, the mechanisms involving suppression of MPB are less well understood. These data would provide targets for future therapeutic adjuvants with or without amino acid infusion to improve muscle amino acid kinetics. Further, our muscle kinetic data are only representative of the perioperative period, we did not assess if this led to an improvement in amino acid kinetics in either the short‐ or long‐term postoperative period. In addition, we did not measure functional outcomes, and therefore cannot state whether improvement in perioperative muscle net balance is effective in restoring patient function or muscle mass.

Future efforts should aim to establish the proper dose of amino acid administration during the perioperative period, as it is possible higher doses would result in improved muscle kinetics. Further, co‐administration of amino acids with other nutrients such as carbohydrates or ketones is a fruitful avenue as both have been shown to improve amino acid kinetics in catabolic stressed states (Sapir et al., 1972; Thomsen et al., 2018). Another consideration would be investigating the continued infusion of amino acids or provide them in an easy to digest format (e.g., free‐form AA or protein hydrolysates) beyond the perioperative period. Lastly, measurement of functional outcomes such as muscle strength and mass in the postoperative period are crucial to determine if perioperative AA infusion is a practice worth adopting. Nonetheless, our data support the use of perioperative infusion of amino acids during surgery as a pragmatic therapy to attenuate muscle protein loss arising from the initial surgical insult.

## CONFLICT OF INTEREST

All authors reported no conflict of interest.

## AUTHOR CONTRIBUTION

RRW and AAF conceived the idea; RRW and AAF planned original experiments; SES and AAF performed the original experiments; DDC and AAF compiled the results and completed the analyses; DDC lead in writing the manuscript; All authors (DDC, SES, RRW, and AAF) provided critical feedback that helped shape the research, analysis and manuscript.
